# Highly robust crystalsome via directed polymer crystallization at curved liquid/liquid interface

**DOI:** 10.1038/ncomms10599

**Published:** 2016-02-03

**Authors:** Wenda Wang, Hao Qi, Tian Zhou, Shan Mei, Lin Han, Takeshi Higuchi, Hiroshi Jinnai, Christopher Y. Li

**Affiliations:** 1 Department of Materials Science and Engineering, Drexel University, 3141 Chestnut Street, Philadelphia, Pennsylvania 19104, USA; 2 School of Biomedical Engineering, Science and Health Systems, Drexel University, Philadelphia, Pennsylvania 19104, USA; 3 Institute of Multidisciplinary Research for Advanced Materials (IMRAM), Tohoku University, 2-1-1, Katahira, Aoba-ku, Sendai 980-8577, Japan

## Abstract

Lipids and amphiphilic block copolymers spontaneously self-assemble in water to form a plethora of micelles and vesicles. They are typically fluidic in nature and often mechanically weak for applications such as drug delivery and gene therapeutics. Mechanical properties of polymeric materials could be improved by forming crystalline structures. However, most of the self-assembled micelles and vesicles have curved surfaces and precisely tuning crystallization within a nanoscale curved space is challenging, as the curved geometry is incommensurate with crystals having three-dimensional translational symmetry. Herein, we report using a miniemulsion crystallization method to grow nanosized, polymer single-crystal-like capsules. We coin the name crystalsome to describe this unique structure, because they are formed by polymer lamellar crystals and their structure mimics liposomes and polymersomes. Using poly(L-lactic acid) (PLLA) as the model polymer, we show that curved water/*p*-xylene interface formed by the miniemulsion process can guide the growth of PLLA single crystals. Crystalsomes with the size ranging from ∼148 nm to over 1 μm have been formed. Atomic force microscopy measurement demonstrate a two to three orders of magnitude increase in bending modulus compared with conventional polymersomes. We envisage that this novel structure could shed light on investigating spherical crystallography and drug delivery.

## Abstract (web summary)

Self-assemblies of polymers to form polymersomes in solution can be used as carriers for drug delivery, but it is challenging to control polymer crystallization to improve their mechanical stability. Here, Wang *et al.* show the formation of nanosized crystalsomes composed of polymer lamellar single crystals.

## Graphical abstract

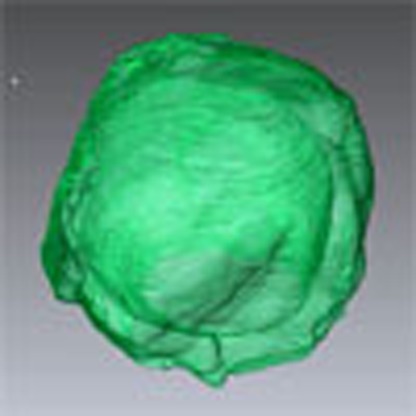

Lipids and amphiphilic block copolymers spontaneously self-assemble in water to form spherical micelles, worm-like micelles and vesicles (also known as polymersome), most of which exhibit curved surfaces^1^,^2^,^3^,^4^,^5^,^6^. Compared with supramolecular assemblies derived from low-molecular-weight lipids (liposomes), these polymer-based assemblies possess a number of distinctive physical characteristics. For example, because of their large molecular weights, polymeric assemblies are mechanically more stable and can sustain their morphologies longer after dilution, which is critical for applications such as drug delivery and gene therapeutics^1^,^7^,^8^. To further increase the kinetic stability of self-assembled capsules, a few alternative strategies have been proposed, including shell/core cross-linked nanoparticles, among others^9^,^10^. In most of these reported ensembles, the structural motives are mobile/liquid in nature. As relatively rigid micelles could enhance the much-needed mechanical stability of the structure, and that many block copolymers preferred for applications are semicrystalline, it is of great interest to tailor polymer crystallization to enhance the kinetic stability of these ensembles^11^,^12^. Morphological evolution and temperature responsive behaviours of semicrystalline block copolymers in solution have also been reported^13^,^14^,^15^. Precisely controlling polymer crystallization in these non-flat ensembles is extremely challenging, because curved space is incommensurate with typical ordered structures having three-dimensional translational symmetry. Colloids have been assembled at curved liquid/liquid interface through a crystallization process^16^,^17^. Dinsmore *et al.*^17^ used this technique to fabricate solid capsules, named as colloidosome, with precise control of size, permeability and mechanical strength. The fundamentals of these two-dimensional spherical crystals are intriguing and some key concepts of crystallography such as grain boundaries, defect formation and elastic instability are interestingly different from those occurring in a flat space^18^,^19^,^20^.

Small crystal grain sizes and large amounts of defects are inevitable. Polymer crystals, on the other hand, have been shown to exhibit non-flat morphology^21^ and their intrinsic high defect density facilitates the formation of such unconventional crystals^22^. We postulate that growing polymer single crystals at curved liquid/liquid interface could serve as a model system to investigate the fundamentals of the above mentioned spherical crystallography^18^,^20^. Herein we disclose a new strategy to grow nanosized polymer single-crystal-like capsules using directed crystallization at curved liquid/liquid interface. In this approach, crystalline polymers are dissolved in an oil phase and then emulsified with an aqueous solution using a miniemulsion approach^23^. Nanosized oil droplets are created, which provides a curved liquid/liquid interface to guide the subsequent polymer crystallization/assembly. Nanosized polymer single-crystal-like capsules (named as crystalsomes) have been formed and they show significantly enhanced mechanical properties compared with liposomes and polymersomes.

## Results

### Emulsion crystallization of poly(L-lactic acid)

**Figure 1:**
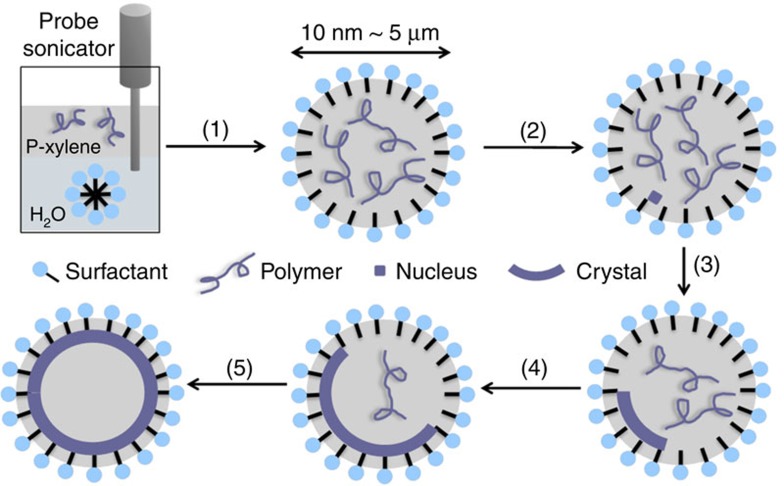
Schematic representation of the fabrication process of crystalsomes. (1) Emulsification; (2) quench to the crystallization temperature; and (3–5) different stages of crystal growth.

We select a crystalline and biocompatible polymer, poly(L-lactic acid) (PLLA), to conduct the experiments. *p*-Xylene and cetyltrimethylammonium bromide (CTAB) are chosen as the oil phase and surfactant, respectively. As Fig. 1 shows, PLLA and CTAB are first dissolved in *p*-xylene and water, respectively. Two solutions are mixed and ultrasonicated to generate a miniemulsion system with PLLA/*p*-xylene droplets stabilized by CTAB in water. The emulsion is then quenched to a pre-determined temperature for crystallization. We envisage that on cooling an emulsion comprising water and semicrystalline polymer solution, three scenarios could occur: (1) polymer solution phase separates within the emulsion droplets;^24^ (2) polymer crystallizes into dendrites/spherulites, filling in the emulsion droplets;^24^ and (3) polymer single crystals form at the water/oil interface. Scenario 3 is the desired pathway in our design, to achieve which, we need to avoid liquid/liquid phase separation of the polymer solution (scenario 1) as well as the dendrite/spherulite formation (scenario 2). Supplementary Fig. 1 shows the PLLA/*p*-xylene phase diagram, according to which liquid/liquid phase separation can be avoided by conducting crystallization above the spinodal line. A very slow crystallization process would favour the formation of single crystals as opposed to dendrites/spherulites. Therefore, 90 °C is chosen as the crystallization temperature; the crystallization rate is relatively slow and the crystals formed at this temperature is relatively isotropic^25^. The latter shall facilitate the formation of a crystalline shell.

**Figure 2:**
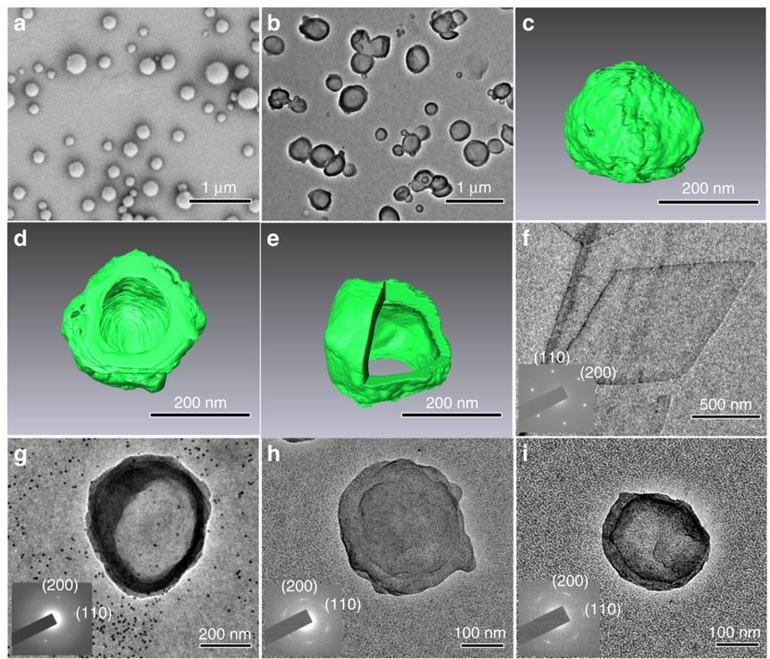
PLLA crystals formed by miniemulsion crystallization. (**a**,**b**) Typical SEM (**a**) and TEM (**b**) images of the PLLA crystalsomes. Side (**c**), bottom (**d**) and top (**e**) view of reconstructed three-dimensional images of the PLLA crystalsomes using transmission electron tomography. In (**e**), the structure is ‘cut' open for the viewing purpose. (**f**) TEM image of a flat PLLA crystal and its corresponding electron diffraction pattern; (**g**–**i**) TEM images of different sized PLLA crystalsomes with their corresponding SAED patterns.

Figure 2 shows the scanning electron microscopy (SEM) and transmission electron microscopy (TEM) images of the PLLA crystals formed following such a crystallization process and the crystallization time was ∼48 h. The crystals are all spherical, although an unconfined PLLA crystal formed in *p*-xylene solution is flat with a lozenge shape^25^. The average diameter of the spherical structures is 255±105 nm, as determined from the image analysis (Supplementary Fig. 2). TEM micrographs (Fig. 2b) suggest that the crystals are hollow, which can be confirmed by removing part of the crystal using a focused ion beam (FIB). As the SEM image of the same crystalsome before and after FIB cutting presented in Supplementary Fig. 3, a hollow inside of the crystalsome can be clearly seen.

### Structure of PLLA crystalsomes

Transmission electron tomography was used to further study the three-dimensional structure of the crystal and the results are shown in Fig. 2c–e. Figure 2c is a side view and Fig. 2d is a bottom view of a reconstructed PLLA spherical crystal^26^,^27^. As the bottom part of the crystal is in contact with the carbon film, substrate, the reconstructed image shows an opening in Fig. 2d. Figure 2e reveals the reconstructed image that had been cut from the top for the viewing purpose. Three-dimensional, hollow structure of the crystal can be clearly seen. The thickness of the shell can be measured to be ∼22.5 nm, corresponding to the thickness of two layers of PLLA single lamellae^28^,^29^,^30^. This can be confirmed by atomic force microscopy (AFM) measurements of the small crystal pieces broken by ultrasonication: their height profile shows a typical thickness of the lamella is ∼11 nm (Supplementary Fig. 4). The movies of a reconstructed PLLA crystalsome can be found in the Supplementary Information. All these experiments suggest that nanosized hollow shells were formed during the miniemulsion crystallization process. Owing to the similarity of our structure and vesicles, and that they are made of polymer lamellar crystals, we use the term ‘crystalsome' to describe this unique structure.

The formation of hollow crystalsomes confirms that scenario 3 did occur in our controlled crystallization process. As shown in Fig. 1, the formation process can be explained by first forming a crystal nucleus in the oil droplet. The nucleus is then pinned at the liquid/liquid interface to lower the global free energy, a phenomenon known as Pickering emulsion^31^,^32^,^33^. Subsequent growth of the polymer crystals is guided by the liquid/liquid interface, leading to crystalsomes. Supplementary Fig. 5 shows the TEM images of the crystalsomes at different growth stages.

To further understand their crystalline structure, selected area electron diffraction (SAED) experiments on individual crystalsome were conducted. Different sized PLLA crystalsomes were prepared by varying the emulsification conditions, which are summarized in Table 1. Samples are labelled as *CS*^*PLLA*^-*n*, wherein ‘*CS*' denotes crystalsome and ‘*n*' is from 1 to 5, representing the crystalsomes with different sizes. For comparison, flat PLLA single crystals were also grown in solution. SAED patterns of all the crystalsomes show the typical PLLA single diffraction pattern as shown in Fig. 2f–i. This is intriguing, because typical polymersomes, owing to their fluidic nature of the ensembles, show spherical symmetric and all the radial directions of the spheres are identical. In the present case, however, despite the spherical shape, crystalsomes are made of crystals with distinctive crystallographic plane/directions and only one crystalline orientation per crystalsome was observed. Also of interests is that as the diameters of the crystalsomes decrease from ∼500 to 314 and 148 nm (Fig. 2g–i), the corresponding diffraction spots change from spot-like to eventually arc shape. This is related to the fact that as the curvature increases for small crystalsomes, it becomes increasingly more difficult for polymer chains to pack in the curved space and follow the three-dimensional translational symmetry—defects therefore have to be introduced for small sized crystalsomes.

**Figure 3:**
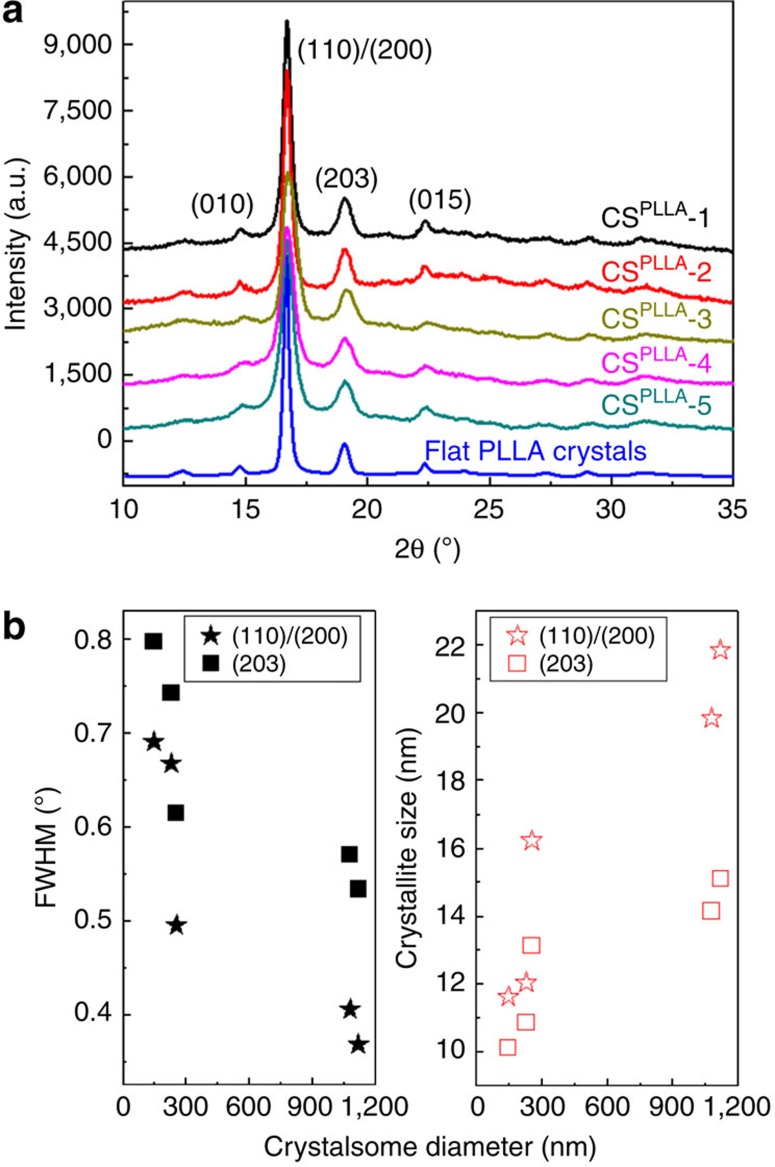
Crystal structures of PLLA crystalsomes. (**a**) WAXD spectra of PLLA crystalsomes and flat PLLA crystals. (**b**) FWHM of (110)/(200) and (203) diffraction peaks, and the corresponding crystallite sizes of various crystalsomes.

Decreased order in small crystalsomes can be quantified using wide angle X-ray diffraction (WAXD) experiments. The WAXD patterns in Fig. 3a show that all the PLLA crystalsomes exhibit peaks at the same diffraction angles compared with the flat PLLA single crystals, indicating that PLLA crystalline structures are not altered by the miniemulsion crystallization process. The strongest peak at 16.7° is from the diffraction of (110)/(200) planes and the peak at 19.8° is from (203). Nevertheless, there are two interesting differences between the WAXD patterns from the flat PLLA crystals and those from PLLA crystalsomes: (1) the amorphous halo is more significant in crystalsomes and the crystallinity decreases with decreasing crystalsome size, that is, from 72% for the flat crystal to 55.3%, 52.6%, 43.5%, 40% and 35% for crystalsomes with diameters of 1,120, 1,080, 255, 230 and 148 nm, respectively. (2) The flat PLLA crystal has the sharpest diffraction peaks among all the samples tested. The full widths at half maximum (FWHM) of each diffraction peak are different: FWHM increases as the crystalsome became smaller, as plotted in the left panel of Fig. 3b. The average crystallite size can be calculated from FWHM using the Scherrer equation and the trend is plotted in the right panel of Fig. 3b. As the size of the crystalsome decreases from 1,120 to 148 nm, the average crystallite size decreases from 22 to ∼11.8 nm. These results are consistent with the crystallinity observation.

### Mechanical properties of PLLA crystalsomes

**Figure 4:**
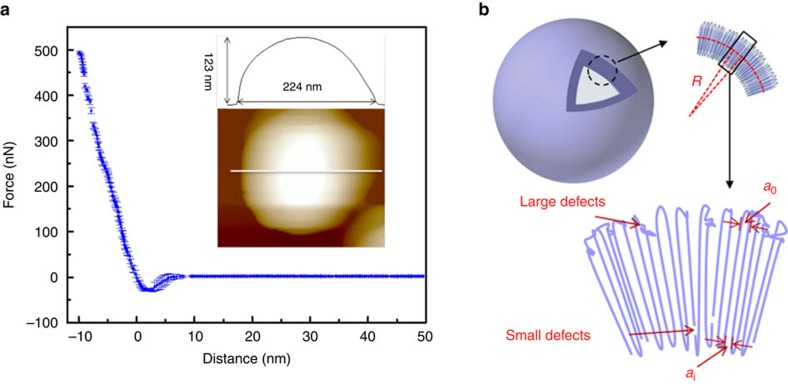
Mechanical properties of crystalsomes. (**a**) AFM force-deformation spectrum on a PLLA crystalsome. Inset: representative AFM image of a single PLLA crystalsome and its corresponding height profile; (**b**) schematic representation of a typical crystalsome and its defect distribution.

The mechanical property of the PLLA crystalsomes was measured using AFM force spectroscopy (a large-scale AFM image is shown in Supplementary Fig. 6). The PLLA crystalsome was deposited on a silicon wafer. Figure 4a shows the AFM image of a PLLA crystalsome acquired under Tapping Mode. The height profile indicates that the height of the crystalsome is 123 nm and the lateral size is ∼224 nm. According to this measurement, the crystalsome is moderately flattened at the bottom on deposition and the upper part still maintains the spherical structure, consistent with the tomography results. The force-deformation curve is shown in Fig. 4b. Accordingly, membrane stiffness can be calculated by linear fitting the small deformation portion of the curve and the slope of the fitted line is the stiffness. The equation relating the stiffness *k*_membrane_ of the membrane with Young's modulus has been derived in the classical shell theory. The solution of deformation of a spherical shell under point loads on its pole can be expressed as equation (1):^34^,^35^


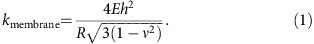


Here, *E* is the Young's modulus, *h* is the shell thickness, *ν* is the Poisson ratio and *R* is the radius of the crystalsome, which can be calculated from the AFM measurements. It is worth noting that the validity of equation (1) is restricted to small deformation^36^. The membrane-bending modulus *K*_bend_ of a capsule with size *R* made with material with Young's modulus *E* can be calculated using equation (2):


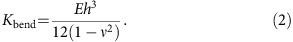


This method has been widely used to study the mechanical properties of single micro- or nano-capsules^37^,^38^,^39^,^40^,^41^, or biological specimens^42^. To obtain the stiffness of a crystalsome, 25 measurements were performed on the crystalsome and calculated stiffness obtained from approaching curves is 46.6±6.5 N m^−1^ (ref. 39). The modulus of the crystalline PLLA is ∼4.16 GPa, calculated from equation (1) using an *R* of 112 nm, *h* of 22.5 nm and a *ν* of 0.36. Plugging the calculated modulus into equation (2), the membrane bending modulus is determined to be 4.54 × 10^−15^ J. This is around 100–1,000 times higher than the reported data for polymersomes^37^,^39^. It is worth noting that the shell thickness of PLLA crystalsomes is quite close to that of the membrane thickness of typical polymersomes and the significant enhancement in mechanical properties can be attributed to the two key features of crystalsomes: (1) they are made of solid crystalline polymers and (2) the packing of polymer chains/defects in the crystalsome. As previously discussed, decreasing crystallinity with decreasing crystalsome size is anticipated, because it becomes increasingly more difficult to pack polymer chains within curved space with high curvature while maintaining the translational symmetry. Defects are induced with decreased crystalsome size, as evidenced by the decreased crystallinity. It is also of great interest to investigate the distribution of the defects. As shown in Fig. 4b, polymer crystals in a curved space/interface have to splay their chains from the inner layer to the outer layer, to compensate the curved geometry. If the distance between the adjacent chain at the very outer layer is *a*_o_ and at the very inner layer is *a*_i_ as shown in Fig. 4b, the relative ‘strain' of the crystalsome between the inner and outer shell can be defined as *ɛ*=*(a*_o_*−a*_i_*)/a*_i_. Assuming the thickness of the crystalline lamella is *l*, *ɛ* can be rewritten as *ɛ*=*2 l/(d−l)*, in which *d* is the diameter of the crystalsome, and as there are two layers of lamellae in each crystalsome in the present case, *l* is the approximated half of the crystalsome thickness *h*. Table 1 shows that at 148 nm, *ɛ* can be up to 14.5%. To compensate this difference, larger defects or even amorphous polymer chains (such as ‘dead knots' due to entanglements) should be excluded to the outer layer, serving as ‘wedges' to facilitate chain packing. Small-sized defects such as vacancies will tend to locate near the inner side of the shell. This unique arrangement of the defects should account for the high bending modulus observed in our system. In addition, it is noteworthy that this packing is feasible only because that the crystalsome was formed via a nucleation–slow–growth process and the defects have sufficient time to arrange and accommodate the curved space at the liquid/liquid interface.

## Discussion

We demonstrate a novel miniemulsion crystallization process where polymer solution is used as the oil phase. Controlled crystallization of this miniemulsion system leads to the formation of nanosized polymer single-crystal-like crystalsomes. SAED and WAXD experiments show that the crystalsomes are made of polymer crystals whose crystallinity decreases with crystalsome sizes. These crystalsomes are similar to the previously reported polymersomes, because they both are enclosed nanosized polymer ensembles. They are also fundamentally different, because the present crystalsomes are made of crystalline homopolymers, whereas polymersomes are formed by amphiphilic block copolymers or homopolymers with both hydrophobic and hydrophilic moieties^43^,^44^; they are grown based on directed growth of polymer single crystals at liquid/liquid interface; and crystalsomes exhibit two to three orders of magnitude higher bending modulus compared with conventional polymersomes. We envisage this novel structure could shed light on fundamental research on crystallization at curved space and polymer self-assembly for drug delivery and gene therapeutics. Future work will be focused on tuning the structure of crystalsomes for drug delivery.

## Methods

### Materials

PLLA (*M*_n_=10,000 Da, polydispersity index (PDI)=1.1), *p*-xylene, CTAB and Gold nanoparticle colloidal solution were purchased from Sigma and were used as received.

### Emulsion crystallization of PLLA

PLLA/*p*-xylene solution (4 wt%) was prepared in a glass test tube at 120 °C and was slowly cooled to 98 °C for emulsification. The solution was mixed with CTAB aqueous solution, which was preheated to 98 °C with the weight ratios in Table 1. After emulsification at 98 °C by 5 min probe sonication at the sonication amplitude indicated in Supplementary Table 1, the emulsions were quenched to 90 °C for crystallization for 48 h.

### Characterization

FIB experiments were conducted on FEI Strata DB235 FIB/SEM with an Omniprobe model 100.7 micromanipulator. The sample was placed on silicon wafer piece and attached to a standard SEM stub. WAXD experiments were conducted on RigakuSmartLab. After crystallization, the emulsion was broken by adding ∼1,000 times of deionized water. Vacuum filtration setup was used to wash away CTAB and a white powder was obtained for WAXD experiments. AFM images and force spectra were acquired using a Bruker Dimension Icon AFM equipped with a tapping mode and PeakForce mode. Samples were dried on silicon wafers. The cantilever we used is Bruker TESPA with tip height 10–15 μm and tip radius 8 nm. The front angle is 25±2.5°, the back angle is 15±2.5° and the side angle is 22.5±2.5°. The average spring constant of *k*=14.38 N m^−1^, calibrated by the thermal tune method. SEM images were taken using Zeiss Supra 50VP. Emulsion products were directly dropcast onto silicon wafer pieces. DI water was used to rinse the silicon wafer to remove CTAB. Before SEM examination, the samples were coated with Pt/Pd. Conventional TEM experiments were conducted on JEOL JEM2100 using 200 KV accelerating voltage. TEM samples were prepared by dropcasting crystallized emulsion onto a Cu grid with a polyvinylformal supporting membrane followed by DI water rinsing. SAED of the shells was also obtained using this instrument with 120 kV accelerating voltage. For the electron tomography experiments, 2 μl crystalsomes in *p*-xylene suspensions were dropped on a Cu grid with a polyvinylformal supporting membrane and dried at room temperature. Gold nanoparticles with 5 nm in diameter were placed on the backside of supporting membrane by dropping 2 μl of their colloid. The electron tomography observation was conducted by a JEM-2200FS (JEOL, Ltd, Japan) operated at 200 kV and equipped with a slow-scan USC 4000 CCD (Gatan, Inc., USA). A tilt series of TEM images were acquired in the range of ±73° at angular interval of 1°. The tilt series were aligned by fiducial marker method by using gold nanoparticles as the fiducial markers^45^. After the alignment carried out by homemade software, the tilt series were reconstructed by filtered back projection algorithm^46^.

## Additional information

**How to cite this article**: Wang, W. *et al.* Highly robust crystalsome via directed polymer crystallization at curved liquid/liquid interface. *Nat. Commun.* 7:10599 doi: 10.1038/ncomms10599 (2016).

## Supplementary Material

Supplementary Information Supplementary Figures 1-6, Supplementary Table 1 and Supplementary Methods

Supplementary Movie 1 Three dimensional view of a reconstructed PLLA crystalsome using electron tomography-rotation-shaded view.

Supplementary Movie 2 Three dimensional view of a reconstructed PLLA crystalsome using electron tomography-rotation-transparent view.

Supplementary Movie 3 Three dimensional view of a reconstructed PLLA crystalsome using electron tomography. The image was artificially “cut” for more clear view.

## Figures and Tables

**Table 1:** Preparation conditions and characteristics of crystalsomes.

**Sample**	**Water-*****p*****-xylene-CTAB**	**Average size (nm)**	**Crystallinity**	***ɛ*** **(%)**
CS^PLLA^-1	80–19.90–0.10	148	35%	14.5
CS^PLLA^-2	80–19.96–0.04	230	40%	9.1
CS^PLLA^-3	70–29.94–0.06	255	43.5%	8.2
CS^PLLA^-4	70–29.94–0.06	1,080	52.6%	1.9
CS^PLLA^-5	70–29.94–0.06	1,120	55.3%	1.8
Flat crystal	—	—	72.0%	0

CTAB, cetyltrimethylammonium bromide.
