# Supplementary material for: Highly robust crystalsome via directed polymer crystallization at curved liquid/liquid interface
Source: Nat Commun. 2016 Feb 3;7:10599. doi: 10.1038/ncomms10599 (PMC4742919; doi:10.1038/ncomms10599)
Supplement: Supplementary Information — Supplementary Figures 1-6, Supplementary Table 1 and Supplementary Methods [file ncomms10599-s1.pdf]

Supporting information,

Supplementary Figures

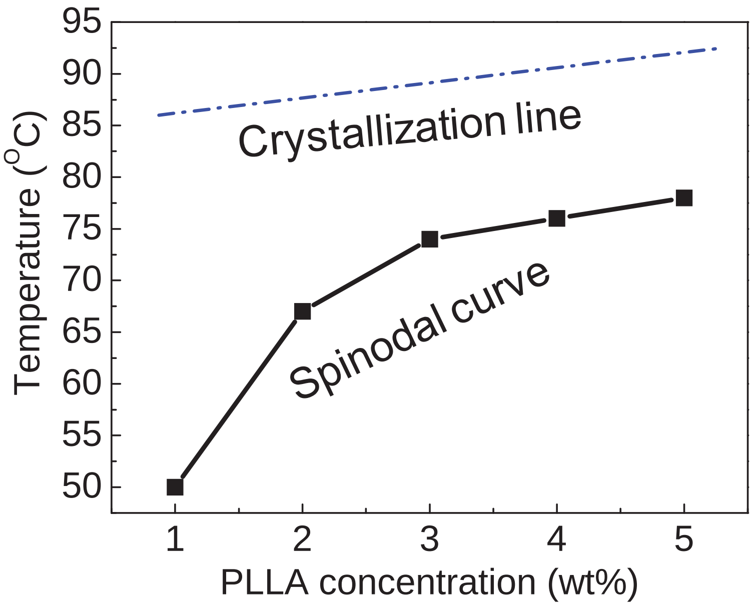

Supplementary Figure 1. PLLA/p-xylene solution phase diagram.

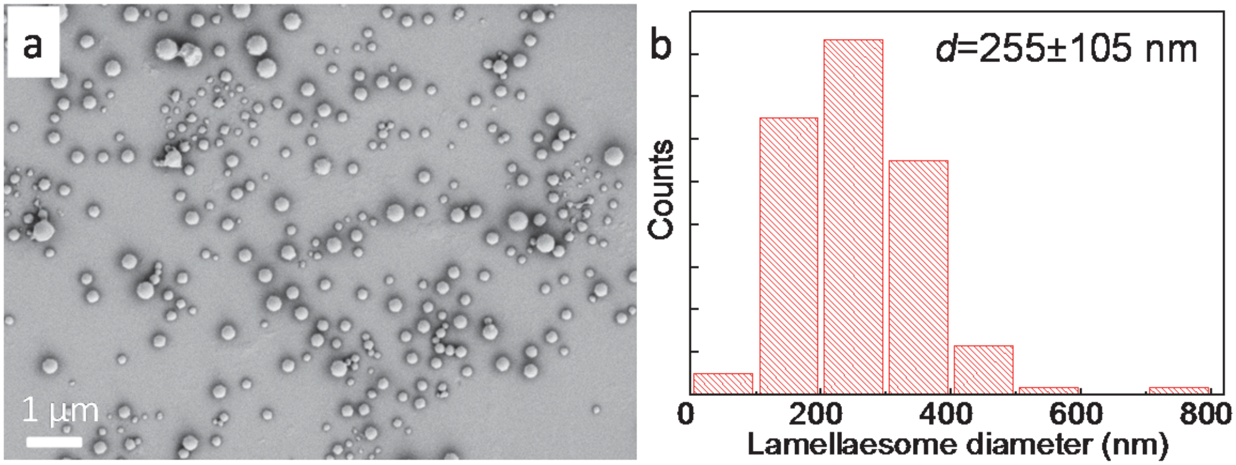

Supplementary Figure 2. (a) SEM image of PLLA crystalsomes; (b) histogram of size distribution PLLA crystalsomes.

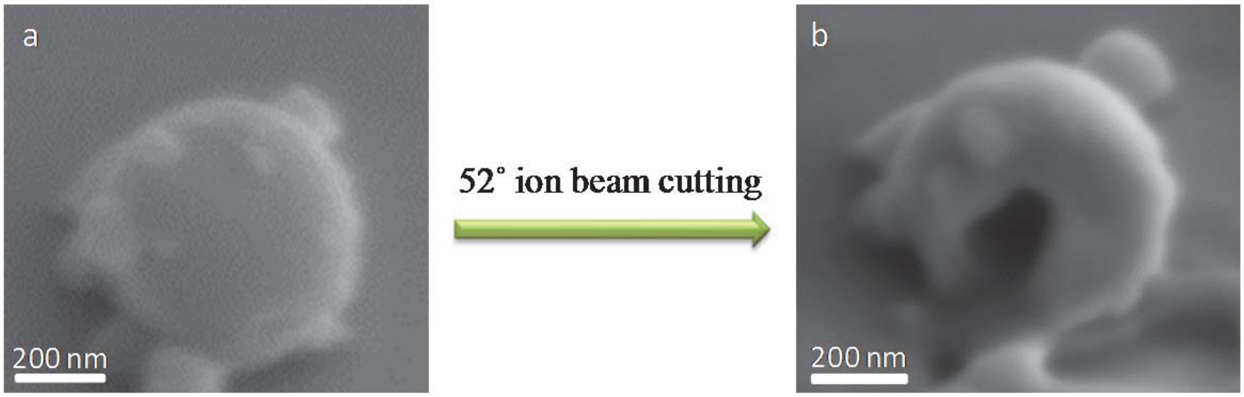

Supplementary Figure 3. SEM images of a PLLA crystalsome before (a) and after (b) focus ion beam cutting.

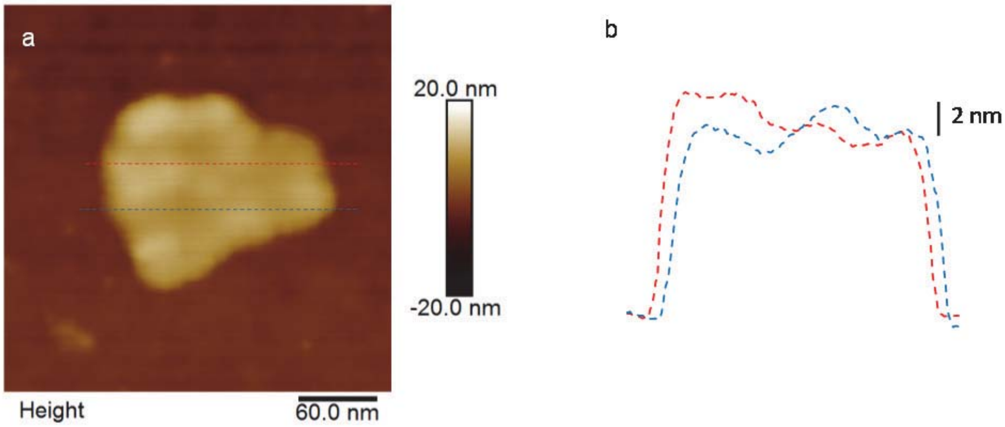

Supplementary Figure 4. (a) AFM images of the small crystal pieces broken by ultrasonication; (b) height profile of the piece drawn from the white line indicated in (a).

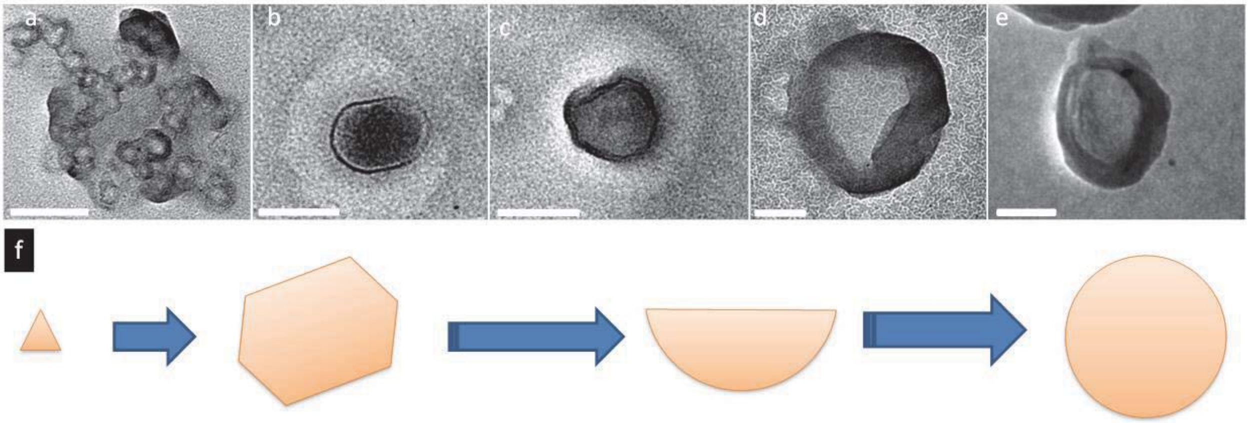

Supplementary Figure 5. TEM images of PLLA crystalsomes at different growth stages. The samples were formed with (a) 0.1 wt%, (b) 0.2 wt%, (c) 0.5 wt%, (d) 2 wt%, and (e) 4 wt% initial PLLA concentration. The emulsification used a 70-29.94-0.06 water-*p*-xylene-CTAB ratio, and the crystallization time was 48h.

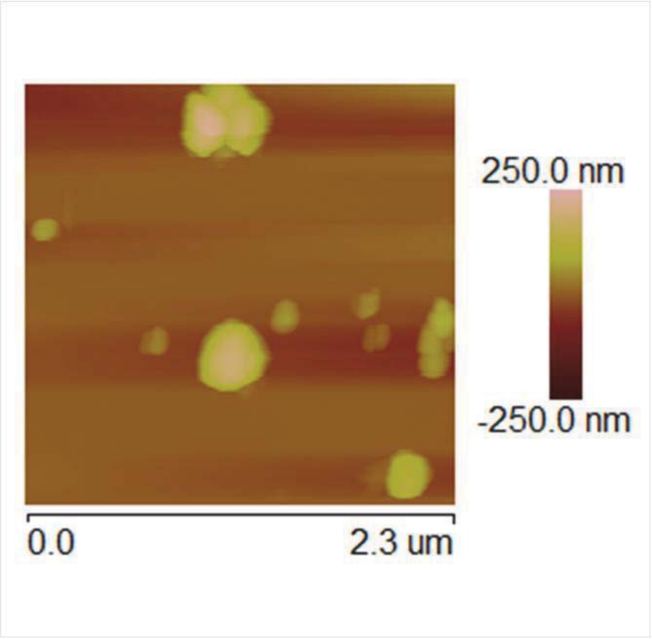

Supplementary Figure 6. AFM image of PLLA crystalsomes.

Supplementary Table 1. Summary of experimental conditions for preparation of the PLLA lamellasomes.

Sample label	Experimental Condition	
	Water-*p*-xylene-CTAB weight ratio	Sonication power
**LS^{PLLA}-1**	80-19.9-0.1	90%
**LS^{PLLA}-2**	80-19.96-0.04	90%
**LS^{PLLA}-3**	70-29.94-0.06	90%
**LS^{PLLA}-4**	70-29.94-0.06	70%
**LS^{PLLA}-5**	70-29.94-0.06	40%
**Flat PLLA Crystal**	Pure p-xylene	No sonication

Supplementary Methods

Calculation of crystallite size using Scherrer equation.

FWHM of each peak is measured after peak fitting using MagicPlot. The crystallite size is calculated using Scherrer equation shown below.

$$\tau = \frac{K\lambda}{\beta \cos\theta} \quad \text{(Supplementary Equation 1)}$$

τ is the crystallite size,

K is a dimensionless shape factor, 0.9 is used in the present calculation,

λ is the X-ray wavelength which is 0.154 nm,

β is FWHM in radian,

θ is the Bragg angle.
